# Recent advances towards tuberculosis control: vaccines and biomarkers

**DOI:** 10.1111/joim.12212

**Published:** 2014-04-18

**Authors:** J Weiner, S H E Kaufmann

**Affiliations:** Department of Immunology, Max Planck Institute for Infection BiologyBerlin, Germany

**Keywords:** bacille Calmette–Guérin, biomarker, biosignature, tuberculosis, vaccine

## Abstract

Weiner 3^rd^ J, Kaufmann SHE (Max Planck Institute for Infection Biology, Berlin, Germany). Recent advances towards tuberculosis control: vaccines and biomarkers. (Review). *J Intern Med* 2014; **275**: 467–480.

Of all infectious diseases, tuberculosis (TB) remains one of the most important causes of morbidity and mortality. Recent advances in understanding the biology of *Mycobacterium tuberculosis* (*Mtb*) infection and the immune response of the infected host have led to the development of several new vaccines, a number of which are already undergoing clinical trials. These include pre-exposure prime vaccines, which could replace bacille Calmette–Guérin (BCG), and pre-exposure booster vaccines given in addition to BCG. Infants are the target population of these two types of vaccines. In addition, several postexposure vaccines given during adolescence or adult life, in addition to BCG as a priming vaccine during infancy, are undergoing clinical testing. Therapeutic vaccines are currently being assessed for their potential to cure active TB as an adjunct to chemotherapy. BCG replacement vaccines are viable recombinant BCG or double-deletion mutants of *Mtb*. All booster vaccines are composed of one or several antigens, either expressed by viral vectors or formulated with adjuvants. Therapeutic vaccines are killed mycobacterial preparations. Finally, multivariate biomarkers and biosignatures are being generated from high-throughput data with the aim of providing better diagnostic tools to specifically determine TB progression. Here, we provide a technical overview of these recent developments as well of the relevant computational approaches and highlight the obstacles that still need to be overcome.

## Introduction

Three major infectious diseases, acquired immune deficiency syndrome (AIDS), tuberculosis (TB) and malaria, are the cause of an enormous disease burden, with high rates of both morbidity and mortality 1–4. Every year, TB alone causes active disease in 8.6 million individuals of whom 1.3 million die 2. The cause of TB is an acid-fast bacillus, *Mycobacterium tuberculosis* (*Mtb*), which has developed a successful survival stratagem 5–7. The pathogen is surrounded by a highly resistant cell wall rich in waxes and lipids 8. Therefore, it can survive for long periods of time, even under highly unfavourable conditions, either in the environment or in the human host. The preferred organ of TB disease manifestation is the lung, which allows spreading of *Mtb* through the most efficacious route: exhalation and expectoration 9. The source of transmission is the patient with active pulmonary TB, the most prevalent form of this disease. Transmission is highly efficient as indicated by the fact that two billion individuals are infected with *Mtb* worldwide. However, the majority of these infected individuals do not develop active disease and are not contagious. Rather, in these individuals *Mtb* remains at a subclinical stage termed latent TB infection (LTBI). Given an efficacious immune response comprising primarily T lymphocytes and mononuclear phagocytes, which orchestrate formation of solid granulomas, this restraint can be lifelong. Within these granulomas, however, *Mtb* persists and becomes dormant (i.e. metabolically inactive and nonreplicative) 8. This can be viewed as a ‘peaceful’ coexistence in which *Mtb* is virtually inaccessible to immune attack and at the same time does not harm its host. However, in 5–10% of LTBI cases, such coexistence at some point becomes a ‘conflict’; vigorous *Mtb* replication results in an enormous bacterial load accompanied by uncontrolled immunity which causes extensive tissue damage. As a result, the granuloma first necrotises and then liquefies, thus becoming the source of pathology, as well as of dissemination and transmission of *Mtb*. Massive destruction of the lung causes the characteristic clinical signs of pulmonary TB 9.

## The immune response in TB

Although the general principles of protective immunity against TB are well established, the fine details remain unclear 10–12. This is due in part to the fact that *Mtb* has not been eradicated completely, in the human host or in animal models. Hence, partial rather than absolute protection is achieved by a productive immune response. Epidemiological data indicate that a proportion of individuals who live for long periods in a highly contagious environment remain immunologically naïve with respect to TB 13–15. This could be interpreted as innate host resistance, which is probably independent of acquired immunity.

Essentially, CD4 T-cells of helper type 1 (Th 1) that activate antimycobacterial activity in mononuclear phagocytes are considered central mediators of protection. Activation is mediated by cytokines including interferon-gamma (IFN-γ) and tumour necrosis factor (TNF) 10,11,16. Recent findings suggest a role for IL-17-producing Th17 cells in TB with an early but transient Th17 burst which apparently contributes to protection whereas long-lasting Th17 activity causes pathology 17–19. An additional role of CD8 T lymphocytes is now generally accepted 20, although the contribution of these cells remains unclear 21. Do they contribute to protection by cytolytic mechanisms mediated by perforin and granulysin, or do they primarily serve as a second source of Th1 cytokines 21,22?

Activated macrophages can control growth of *Mtb* but generally cannot fully eradicate the pathogen in various *in vitro* and *in vivo* systems. Reactive nitrogen intermediates generated by the inducible NO synthase are highly potent effectors against *Mtb* in the mouse system 10,11,16,23,24. However, their low abundance in humans raises questions about the importance of their role in human TB. Although direct killing of *Mtb* by granulysin has been observed 22, its relevance *in vivo* requires experimental verification. Further investigation of the immune mechanisms relevant to protection is therefore warranted. The contributions of various types of T-cells (invariant natural killer, gamma-delta, lipid-specific CD1-restricted and unconventional CD8 T-cells, such as mucosal-associated invariant T-cells) may be small 10,11,16,21. Nevertheless, these cells could be important at the cellular level. Similarly, macrophage-activating mediators other than IFN-γ and TNF, including granulocyte–macrophage colony-stimulating factor (GM-CFS), IL-1 cognates and vitamin D derivatives, which have all been shown to stimulate antibacterial defence mechanisms, need to be considered at the humoral level 10–12,25–27. This is particularly noteworthy because IFN-γ fails to stimulate mycobacterial growth inhibition in human macrophages *in vitro*. The complexity and partial redundancy of the immune response in TB presents a barrier against a full understanding of the mechanisms involved. A further complexity is the profound contribution of the immune response to pathology. Regulatory T-cells, which produce IL-10 and transforming growth factor beta (TGF-β), have been identified in experimental animal models and in patients with TB 10–12. The outcome of manipulating such cells is difficult to predict and could affect both protection and pathology. This also holds true for other immune regulatory mechanisms.

A further complicated issue that needs to be considered is the focus of the immune response in TB on granulomatous lesions. Solid granulomas successfully contain *Mtb* whereas caseous granulomas allow them to bloom 8. Accordingly, the three-dimensional tissue organization, which is very difficult to analyze under *in vitro* conditions, plays a significant role. Moreover, increasing evidence suggests that in patients with active TB, different stages of granulomas coexist, which form a spectrum ranging from solid granulomas to cavitary lesions 11,28,29. As a consequence, different host mechanisms coexist, from protective to detrimental, in a single patient with TB in a spatial framework that is hard to decipher using conventional approaches analyzing peripheral blood leukocytes. This high degree of autonomous and dynamic immune response in different regions of the affected lung makes it difficult to conceptualize a generalized vaccination strategy. It is clear that biomarkers that serve as reliable correlates of protection or of pathology would be extremely useful.

In sum, the immune response in TB appears to be a ‘double-edged sword’ that is controlled at various stages. The aim of conventional approaches towards novel vaccination strategies in TB was to mimic/improve immunity caused by natural *Mtb* infection 6. This can lead eventually to vaccines that prevent TB disease but do not achieve sterilizing immunity. Such an immune response can be compared to protective immunity operative in the majority of *Mtb*-infected individuals who remain at the stage of healthy LTBI throughout their lifetime. It could be argued that those who develop TB disease and hence those who are the prime targets of vaccination fail to develop such a protective response comprising the immune mechanisms described above. Consequently, the chances of successfully developing a highly efficacious vaccination strategy based on current approaches would appear to be low.

Efficacious vaccines against infectious diseases in use today are all based on stimulating pre-existing antibodies which neutralize the pathogen or its product soon after infection. Because of the intracellular location of *Mtb*, antibodies have been attributed a minor role in control of TB. It is generally assumed that *Mtb* is already hidden inside mononuclear phagocytes when B-cell-derived plasma cells produce specific antibodies 6,11. In contrast to natural *Mtb* infection, pre-existing antibodies stimulated by prior vaccination could directly attack *Mtb* at the port of entry and thus contribute to vaccine efficacy. This option has thus far been largely ignored in TB vaccine design 30.

### Antigens

Equally perplexing is the issue of protective antigens in TB. In many viral systems, a few dominant antigens are sufficient and essential for protective immunity. In TB, some proteins may be ignored by the immune system but many are recognized resulting in a medium level of activation of the respective antigen-specific T-cell clone(s) 6,31,32. Hence, unique protective T-cell antigens have not been identified thus far. Rather a broad set of antigens exist, which stimulate a measurable but not a striking T-cell response. It is possible that some of these protein antigens contain subdominant or masked epitopes, which are not accessible to the antigen presentation machinery or are concealed by dominant epitopes, respectively 32.

A multigene family comprising nearly 100 genes has been identified in the *Mtb* genome, which is characterized by a proline–glutamine (PE) sequence, and a similar one characterized by a glycine–alanine-rich region known as a polymorphic GC-rich repetitive sequence (PGRS) 33,34. The considerable variability of similar proteins could deviate antigen-specific T-cell responses towards irrelevant antigens, thus resulting in ineffective immunity. Finally, it has been speculated that known T-cell epitopes of *Mtb* are counterproductive for vaccine-induced immunity 31. Most of these epitopes are located in conserved regions and hence could be maintained during evolution to benefit *Mtb* rather than the host. Consequently, epitopes from variable regions should be exploited for future vaccine candidates. Evidence from other infectious diseases, notably malaria and AIDS, supports this assumption 35,36.

## The current vaccine BCG

Bacille Calmette–Guérin (BCG) is an attenuated strain of *M. bovis*, the agent that causes cattle TB. Albert Calmette and Camille Guérin discovered BCG following passage of *M. bovis* to select for attenuated mutants. Indeed after 230 passages, a vaccine strain was created, which had lost its pathogenicity/virulence but was still immunogenic in experimental animals and even in cows (the natural host of *M. bovis*) 37. The vaccine was developed to prevent serious forms of TB in infants, as this was a major cause of neonatal mortality in Europe in the early 20th century. At that time, it was generally expected that approximately a quarter of babies born into a household with a patient with active TB would die, in many cases due to TB. In 1921, the vaccine was administered to a human for the first time. A baby born in a household with a TB patient was vaccinated and grew up without developing TB disease. Subsequently, more than 20 000 infants from households including at least one patient with TB were vaccinated with BCG; the results were impressive as less than 1% of the vaccinated infants died of TB. Although a formal control group was not included in the study, this was a clear reduction in deaths 37. To date, at least four billion doses of BCG have been administered, and as part of the expanded programme on immunization (EPI), its global coverage has reached more than 80%. It is safe, although it imposes a risk for immune-deficient individuals; this led the World Health Organization to recommend that human immunodeficiency virus (HIV)-positive newborn babies should not be vaccinated against BCG 38. Thus, the vision of Calmette and Guérin has become reality. However, protection afforded by BCG is transient, lasting only for a few years. BCG does not provide protection against pulmonary TB in adolescents and adults, which is the most prevalent form of this disease today 39. Hence, better TB vaccines are needed for satisfactory control of this devastating disease.

During their studies in the 1920s, Calmette and Guérin observed that BCG had a broader beneficial effect on childhood mortality: amongst the more than 20 000 vaccinees, only 5% died (including 1% due to TB). This was unexpectedly low compared to the overall mortality in this population of about 25% (see above) 37. Although this led to some speculation at the time about nonspecific protective effects of BCG, it was not pursued further until more recently when epidemiological evidence was presented that BCG can confer general resistance against some communicable and perhaps even some noncommunicable diseases that are under the control of the immune system. Even though the underlying mechanisms are far from understood, the epidemiological data in support of this notion are compelling 40.

Three possible explanations for these effects have been suggested 40. First, a shift from Th2 immunity (which suppresses protective immunity against bacteria and viruses, and is responsible for allergic diseases), towards Th1 immunity (which is critical for protection against viral and bacterial infection) is stimulated. Secondly, cross-reactive T-cell responses are induced, which provide broadly reactive immunity against various pathogens through shared epitopes. Thirdly, long-lasting activation of mononuclear phagocytes occurs, which has recently been termed ‘trained immunity’.

The occurrence of nonspecific activation of mononuclear phagocytes over restricted time periods has long been known. Recently, circumstantial evidence has been provided for long-lasting macrophage activation due to epigenetic alterations 40.

## The TB vaccine portfolio

**Figure 1:**
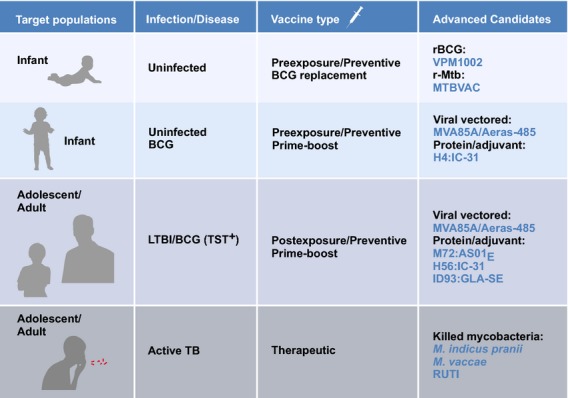
Overview of different vaccine types. Advanced vaccine candidates for different target populations, with stage of vaccine administration.

The main focus of the current portfolio of TB vaccines is prevention of active TB with a few candidates being developed for therapy of TB disease as an adjunct to chemotherapy. Figure1 and Table1 provide an overview of advanced vaccine candidates, their target populations and timing of administration. The majority of preventive vaccines build on immunity induced following priming with BCG. These booster vaccines are either viral vectors expressing one or more *Mtb* antigens, or protein–adjuvant formulations comprising fusion proteins of up to four *Mtb* antigens. Currently, most of these vaccines are designed for postexposure vaccination of adolescents or adults with LTBI who had been vaccinated with BCG in childhood 5–7. Nevertheless, pre-exposure vaccination of BCG-vaccinated infants remains an option.

**Table 1:** Preventive tuberculosis vaccines

Vaccine	Status
VPM1002 (rBCGΔureC:hly)	Phase IIa ongoing in infants
MTBVAC (rMtbΔPhoPΔFadD26)	Phase I ongoing in adults
MVA85A (MVA expressing Rv3804)	Phase IIb completed (no efficacy) in infants
H1 (Rv1886 + Rv3875 fusion protein in adjuvant IC31 or CAF01)	Phase I completed in adults
H4 (Rv1886 + Rv0288 fusion protein in adjuvant IC31)	Phase I completed in adults
MVA85A	Phase IIb ongoing in adults
Ad5HUAG85A (Human Ad 5 expressing Rv3804)	Phase I completed in adults
M72 (Rv1196 + Rv0125 fusion protein in adjuvant AS01)	Phase IIa completed in infants and adults
H56 (Rv1886 + Rv3875 + Rv2660 fusion protein in adjuvant IC31)	Phase I ongoing in adults
ID93 (Rv2608 + Rv3619 + Rv3620 + Rv1813 fusion protein in adjuvant GLA-SE)	Phase I ongoing in adults

See text for further details.

The viral vector–based vaccine candidates include MVA85 Aeras 485 developed by the University of Oxford. This vaccine has recently completed a phase IIb trial in BCG-vaccinated infants, which revealed an excellent safety profile but failed to prove protective efficacy in this population 41. Modified vaccinia Ankara (MVA) induces strong CD4 Th1 cell responses and serves as vector to express antigen 85A, a member of the antigen 85 family, which is used for numerous vaccine constructs (see Table2) 41. A phase IIb trial of MVA85 in BCG-vaccinated HIV-positive adults is ongoing 42. Another viral vector–based vaccine is Crucell Ad35/Aeras 402, which was being investigated in a phase IIb trial in BCG-vaccinated HIV-positive adults and BCG-vaccinated infants; however, the trial was revised to phase IIa with a smaller number of participants probably due to lack of evidence for efficacy 42. This construct is based on adenovirus serotype 35, which expresses three *Mtb* antigens: 85A, 85B and TB10.4 (see Table2). A less advanced viral vector–based vaccine is the antigen 85A-expressing type 5 adenovirus, a phase I trial of which has just been completed 43. Adenoviruses are potent CD8 T-cell stimulators, whereas MVA virus preferentially stimulates IFN-γ-producing CD4 Th1 cells.

**Table 2:** Antigens included in protein–adjuvant-formulated and viral vector–based TB vaccines

Rv number	Generic name	Stage of *Mtb* ‘life cycle’	Vaccine candidate	Comment
Rv3804	Ag85A	Active *Mtb*	MVA85A, Crucell Ad35, Ad5HUAG85A	Fibronectin-binding protein shared by *Mtb* and BCG
Rv1886	Ag85B	Active *Mtb*	H1, H4, H56, Crucell Ad35	Fibronectin-binding protein shared by *Mtb* and BCG
Rv3875	ESAT-6	Active *Mtb*	H1, H56	Early secreted antigenic target
Rv0288	TB10.4	Active *Mtb*	H4, Crucell Ad35	Low-molecular-weight protein. Present in *Mtb* but not in BCG
Rv1196	–	Active *Mtb*	M72	PPE family member shared by *Mtb* and BCG
Rv0125	–	Active *Mtb*	M72	Serine protease pepA shared by *Mtb* and BCG
Rv2660	–	Dormant *Mtb*	H56	Expressed by *Mtb* under starvation conditions. Shared with, but not immunogenic in, BCG
Rv2608	–	Active *Mtb*	ID93	PE-PPE family member shared by *Mtb* and BCG
Rv3619	–	Active *Mtb*	ID93	Esx protein family member shared by *Mtb* and BCG
Rv3620	–	Active *Mtb*	ID93	Esx protein family member shared by *Mtb* and BCG
Rv1813	–	Dormant *Mtb*	ID93	Expressed by *Mtb* under starvation conditions. Shared by *Mtb* and BCG

BCG, bacille Calmette–Guérin; *Mtb*, *Mycobacterium tuberculosis*. For further details on Mtb proteins, see Ref. 87; http://www.tbdb.org/ and http://tuberculist.epfl.ch/.

A large platform of protein–adjuvant formulations has been developed by the Statens Serum Institute, including the hybrid (H) 1 comprising antigen 85B and ESAT-6 with the adjuvants IC31 (H1:IC31) or CAF01 (H1:CAF01) (see Tables2 and 3) 44,45. H1:IC31 is being investigated in a phase IIa trial 42,46 whereas the phase I trial with H1:CAF01 is still ongoing. It is likely that the H1 vaccines will be phased out and succeeded by H56 in IC31 adjuvant, which has completed phase I and is moving into phase IIa development. The H56 vaccine comprises an additional antigen, Rv2660, fused to H1 47. This antigen is preferentially expressed in LTBI and hence H56:IC31 may be suitable for postexposure vaccination of adolescents and adults with LTBI 47. Finally, H4:IC31/Aeras 404 composed of antigen 85B and TB10.4 is being further developed in collaboration with Sanofi Pasteur for vaccination of infants 42; a phase I trial has been completed. The vaccine antigens 85A, 85B, ESAT-6 and TB10.4 are preferentially produced by metabolically active *Mtb* organisms whereas the antigen Rv2660 is a so-called dormancy-related antigen (Table2) 42. The most advanced protein–adjuvant formulation with the adjuvant AS01E has been developed by GlaxoSmithKline and named M72 (Tables2 and 3) 48,49. The M72 fusion protein comprises two antigens typically expressed in active TB disease (Table2). Four different antigens encompassing both active and latent *Mtb* stages have been combined in the ID93 fusion protein, developed by the Infectious Disease Research Institute (IDRI) 50. This fusion protein with the adjuvant GLA-SE is currently undergoing phase I testing (Tables2 and 3).

**Table 3:** Adjuvants used for current vaccine candidates

Adjuvant	Characteristics	Formulation	Vaccine	Source
IC31	Cationic antimicrobial peptide and TLR-9 ligand	KLKL_5_KLK polypeptide and oligodeoxynucleotides	H4, H1, H56	IC
CAF01	Liposome-based, lipoid MINCLE ligand	DDA and TDB	H1	SSI
AS01E	Liposomal-based, surface-active saponin and TLR-4 ligand	Saposin QS21 and MPL	M72	GSK
GLA-SE	Stable emulsion of a TLR-4 agonist and antigen	GLA-SE containing squalene	ID93	IDRI

DDA, dimethyldioctadecyl ammonium bromide; GLA-SE, glucopyranosyl lipid adjuvant stable emulsion; GSK, GlaxoSmithKline; H, hybrid; IC, intercell; IDRI, Infectious Disease Research Institute; MINCLE, macrophage inducible C-type lectin; MPL, monophosphoryl lipid A; O/W, oil in water; SSI, Statens Serum Institute; TLR, Toll-like receptor; TDB, trehalose 6,6′-dibehenate.

Finally, a preparation of killed *Mycobacterium vaccae*, an atypical mycobacterium sp., has been prepared as a booster vaccine and is due to be tested in a phase I trial in the near future 42. This vaccine, DAR-901, is a further development of an *M. vaccae* vaccine originally intended for TB therapy in HIV-positive adults 51. A phase III trial with this vaccine generated data that were difficult to interpret 42 (Table4).

**Table 4:** Therapeutic TB vaccines

Vaccine	Status
*M. indicus pranii*	Retrospective analyses of almost 29 000 phase III study participants. Ongoing phase III trials. Licensed for therapeutic use in certain patients with TB in India
*M. vaccae*	Phase IIb trial completed: no evidence of benefit of *M. vaccae* immunotherapy in patients with TB (retrospective analysis, Cochrane collaboration)
RUTI	Phase IIa trial in LTBI completed
Dar-Dar	Phase III trial in HIV and patients with TB terminated
M72-AS01	Phase IIa trial in patients with TB completed

HIV, human immunodeficiency virus; LTBI, latent tuberculosis infection; *M., Mycobacterium*; TB, tuberculosis.

Amongst the two live vaccine candidates currently in clinical trials, VPM1002 is the most advanced, with a phase IIa trial almost completed successfully in infants, the ultimate target population of this vaccine candidate 52. This vaccine is based on a recombinant BCG with a deletion in urease C and expression of listeriolysin 53,54. The second viable vaccine, MTBVAC, is a double-deletion mutant of *Mtb* and is currently undergoing a phase I trial. It is clear that the purpose of these two viable vaccines is the replacement of BCG and hence they are targeted at pre-exposure vaccination 55.

Some of the current preventive vaccines could also be considered for therapeutic purposes, and M72/AS01E has already been tested in a phase IIa trial in patients with active TB 42. Other therapeutic vaccine candidates are based on whole killed mycobacteria or semi-purified bacterial preparations given as adjunct to chemotherapy 56 (Table4). A member of the latter group is RUTI 57. This vaccine is also considered for prime–boost vaccination in adolescents or adults with LTBI (postexposure vaccination). Phase IIb testing of the vaccine is awaited. *M. vaccae* developed by the Chinese pharmaceutical company, AnHui Longcom, was considered for TB therapy, but careful analysis of available data does not provide evidence of a beneficial effect of this vaccine 42,58. *Mycobacterium indicus pranii*, developed in India, was originally intended as a vaccine against leprosy, but retrospective analysis of a phase III trial provided evidence of protection against TB. A killed preparation of the *M. indicus pranii* vaccine has already been licensed for specific TB cases and is being further evaluated in phase III trials 42,52.

## High-throughput and multiplatform biomarkers in TB

Biomarkers are unique indicators of biological processes 59, and are often applied as surrogate end-points that are used instead of a clinical end-point. Thus, in the case of TB, a potential biomarker should reflect the transition from LTBI to active TB diagnosed by clinical and laboratory means, such as night sweating, cough, expectoration of acid-fast bacilli and lung X-ray 60. Over the last two decades, there have been numerous advances in the field of high-throughput (HT) biology. It is now possible to screen samples for thousands or even hundreds of thousands of variables, including, for example, mRNA, microRNA and protein expression, cytokine levels and metabolites. The generated datasets can then be screened for markers (or predictors) of a particular condition or response. However, the sheer number of variables can generate a new level of complexity for the analysis (for review, see 61).

Generally, the performance of a single biomarker can be markedly increased using instead a tailored combination of independent biomarkers 62. Such a combination is sometimes referred to as a ‘biosignature’ (although this term is frequently used in the context of detecting signs of life or living organisms in general). This principle can be best illustrated by a simple, yet persuasive thought experiment. In a committee that makes decisions based on majority vote, each member has a different probability of making an error. For example, one member of the committee, an expert, errs only once every 100 times, and two other members are wrong once every 20 times. However, even though these two members perform much worse than the expert, they nonetheless contribute to the performance of the committee as a whole, leading to a counterintuitive result: the error rate of the whole committee is 0.3% – three times lower than the error rate for the expert alone. On the rare occasions that the expert is wrong, the two other members have a 90% chance of mitigating the expert decision.

**Figure 2:**
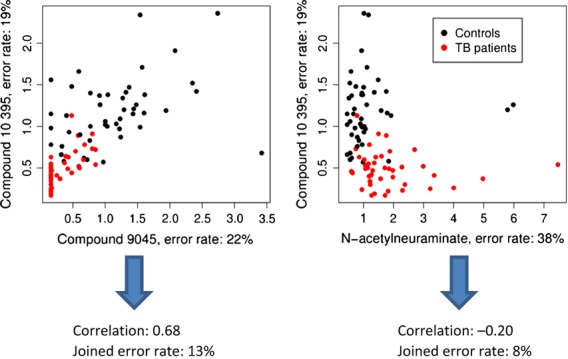
Classification of serum samples (patients with TB and control subjects) using independent predictors. Combining a predictor with an error rate of 22% with a correlated predictor results in a higher joined error rate (13%) than combining the same predictor with an inferior predictor which is not as strongly correlated (joined error rate 8%). Data from Ref. 61.

One important assumption in this example is the independence of the opinions. If, for example, the two nonexpert members always make the same errors as the expert, the performance of the whole committee will clearly not be better than that of the expert alone. Similarly, only when variables within a biosignature are independent, can better performance be expected. It is therefore preferable to select two genes that are not only good predictors but also functionally unlinked (Fig.2).

It is noteworthy that dependence is a broader concept than correlation, particularly if the latter is used in the narrow sense of a Pearson or Spearman correlation coefficient 63. While correlated variables are necessarily dependent, the reverse does not hold true; uncorrelated variables can still lack independence. In the simple example of *x* and *y *= *x*^2^, the variables *x* and *y* are clearly dependent but are not correlated.

Three examples illustrate the ability of tailored biosignatures to improve performance. First, Gibot *et al*. 64 applied a combination of three biomarkers for diagnosis of sepsis: plasma concentrations of soluble triggering receptor expressed on myeloid cells-1 (sTREM-1), plasma concentrations of procalcitonin (PCT) and the expression of Fc fragment of immunoglobulin gamma receptor I (Fc-γRI, CD64) on polymorphonuclear cells. All three components of the new biosignature have previously been proposed as biomarkers. Using a combination of all three biomarkers, it was possible to generate a composite, three-dimensional biosignature which was superior to any of the three single biomarkers.

Secondly, Furman *et al*. 65 profiled gene expression, serum cytokine levels, *in vitro* cytokine stimulation as well as other parameters in search for a biosignature correlated with the specific antibody response after influenza vaccination. Using a supervise machine-learning algorithm (elastic nets, see 66), they identified a set of nine variables that perform well in predicting the response.

**Figure 3:**
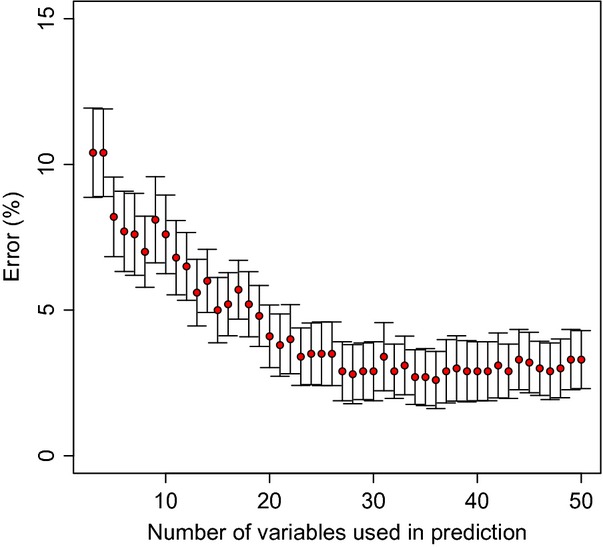
Number of variables selected as predictors versus error rate in a classification task. The error rate decreases with the number of variables that have been chosen for classification. However, adding more than 25 variables to the biosignature does not result in a further improvement of the error rate. Figure from Ref. 61.

Thirdly, in TB, we analyzed metabolic profiles in search of a biosignature capable of correctly discriminating serum samples of patients with TB from serum samples of healthy control subjects 61. Using a bootstrapping approach, we were able to show that the overall classification error rates decrease from about 8 to 12% for biosignatures with less than five variables to about 3% for a biosignature generated with 20–25 variables. Further expansion of the biosignature did not improve the accuracy of this approach (Fig.3).

## Biomarkers in TB

Several studies have focused on biomarkers in TB 67,68. Generally, patients with TB were compared to clinically asymptomatic control subjects, although some studies included as controls individuals with other diseases (contagious and noncontagious diseases such as sarcoidosis) 67–73. It is now well established that patients with TB can be discriminated from healthy control subjects using biomarkers from different platforms, including cytokines (for a review, see Walzl *et al*. 74), gene expression 67–70, proteins 75 and biochemical compounds 61; see also 76. Several biomarkers as well as tailored biosignatures have been identified; indeed, some biomarkers were identified in several studies, including Fc-γRI (CD64) and genes associated with type I IFN signalling. However, it is unclear to what extent these signatures are specific for TB. For example, it has been proposed that Fc-γRI is a general marker of infection 77. Comparisons with other diseases 67,69,71,78,79, including infectious diseases, cancer and sarcoidosis, revealed that biosignatures can be designed to reliably discriminate between different diseases. However, the performance of these biosignatures is still impaired by a high error rate (around 10%; for comparison, see 78).

Biomarkers monitoring drug treatment are of particular interest due to their potential to provide secondary end-points for treatment outcome (such as relapse or success). Global gene expression analyses at the levels of both transcription (mRNA) and translation (protein) indicate however that the most discernible effects are found within the intensive phase of treatment or even in the first 2 weeks 78,80. The applicability of such biosignatures as surrogate end-points becomes questionable if biomarkers fail to reveal significant changes at later treatment time-points.

## How to create a biosignature

A biosignature is more than the enumerating of the names of the variables (predictors) thought to predict a given classification or continuous response. In addition to these variables, it also includes the specific mathematical operation of transforming them into a specific prediction. When applied to new data (a test dataset), the performance of a model can be evaluated; any errors or misclassifications can be used to calculate parameters such as general error rate, accuracy or area under the receiver operating characteristic (ROC) curve. Currently, biomarkers or biosignatures are often used in a setting in which the response is (i) categorical rather than continuous and (ii) often bimodal (i.e. only two possible categories). For a clinical diagnosis, the ideal test result is either positive or negative. In a real-world context, stages of disease with different pathologies are more likely to produce several categories corresponding to the different pathologies.

HT data allow rapid characterization of a large number of samples. Yet frequently the number of samples is much smaller than the number of variables or features (e.g. mRNAs, proteins or metabolites analyzed). Simple frequentist statistical approaches, such as linear regression or anova, will fail. The large number of variables implies a large number of parameters that have to be estimated from an insufficient number of samples. Thus, a reduction of the dimensionality of the data will be required 81.

Perhaps the most straightforward approach is an unbiased removal of some variables prior to the main analysis. This type of dimensionality reduction is known as feature extraction. For example, in the case of microarrays, features that show a low variance or low interquartile range are often removed to reduce the total number being analyzed 82.

Clearly, it is tempting to extract features based on the differences between conditions to be differentiated. For example, to select putative biomarkers for TB, one might want to focus on the set of genes that are differentially expressed between those conditions. However, this procedure cannot be applied outside of model training and validation. In any sufficiently large set of random variables, some will correspond to the response; artificially enriching this set prior to model building using such a ‘specific filtering’ procedure will result in optimistic and incorrect error estimates. Information on the response must not be used as an explorative step before the model has been generated using a cross-validation approach. Variables may be filtered before applying a machine-learning algorithm, but this filtering must be nonspecific and not dependent on the response that the algorithm is supposed to model.

Another way of dealing with high dimensionality is to apply a dimension reduction technique, of which there are several types. Principal component analysis (PCA) is a simple but powerful mathematical procedure in which the bulk of the variance represented by the different variables can be recovered from a few principal components 81. Each component is a linear combination of all or, in variants such as sparse PCA, only a few selected variables. Therefore, interpretation of the principal components and selection of the most important variables are relatively straightforward. Notably, PCA does not rely on the response variable (i.e. disease state). Accordingly, clusters corresponding to the response are unbiased and not a result of applying knowledge *a priori*. A further intriguing feature of PCA is that the components themselves are not correlated. Whilst this in itself does not guarantee independence, it can be sufficient for an accurate and reliable biosignature.

Numerous other dimensionality reduction techniques exist. Some, like PCA, rely on linear transformations of the data whereas others, such as elastic maps 66 or auto-encoders, are inherently nonlinear. Furthermore, some methods [such as sparse PCA or sparse partial least squares (SPLS)] allow the number of variables included in some of the components to be explicitly limited.

Such an approach has been applied recently by Rousu *e al*. 83, who analyzed two sets of data: (i) proteome data from 412 samples from patients with TB and symptomatic as well as asymptomatic control subjects and (ii) plasma proteome profiles from 944 malaria samples from various diagnostic classes (including cerebral malaria, severe malaria anaemia, uncomplicated malaria and community controls). Both datasets included several clinical variables. The aim of the study was first to test the feasibility of the methods used for classification and, secondly, to correlate these with HT variables. Clinical and proteomic datasets were used separately to predict the classification of the samples. Furthermore, proteomic data were used for selection of clinical variables to construct a model based on clinical variables alone; thus, proteomic data were utilized to improve the model at the time of model training, but were not required at the time of making new predictions. Here, the variables were treated as a ‘black box’, without linking the results to the underlying biology. The results showed that selection of clinical variables guided by proteome analysis was able to improve classification accuracy in most but not all comparisons.

Finally, a third way of reducing dimensionality of the data involves understanding the biology of the data. Variables can be grouped according to their biological functions, annotations or chemical properties, as well as experimentally determined functional associations. Gene expression data are often grouped based on functional or cellular localization of gene products, for example using their gene ontology (GO) annotations. Alternatively, it is possible to form groups of co-regulated genes based on an *a priori* experiment in which correlations of gene expression between genes were determined in a range of individuals 65.

For classification tasks and for extracting a biosignature, a range of supervise machine-learning algorithms is available, including support vector machines, elastic nets, partial least squares discriminant analysis (PLS-DA), neural networks and others. These techniques essentially exploit a training dataset, such as gene expression data, to find a model fitting the given response (e.g. classification of health or disease). Several tools of this type have been applied to generate biosignatures of TB 61,67,69,70. As yet, the generated biosignatures are of limited compatibility and often do not perform satisfactorily when selected variables are used in connection with other techniques 78.

It remains debated to what extent the number of variables available from an HT analysis should be reduced. Gene expression studies of host responses in active TB revealed biosignatures based on as few as four genes 84 and up to as many as 393 67. While a larger number of correct variables can improve the performance of a given biosignature, it can also result in the well-known statistical phenomenon of overfitting. At the same time, a rise in the number of variables can increase the chances of including independent variables, thus improving performance.

## Model validation

Biomarker models are frequently based on a large number of variables. Consequently, care must be taken to ensure that models are not overfitted, resulting in an overly optimistic error estimate and that data used to generate the model (the training set) are clearly separated from data used to validate or test the model (the test set).

Due to the large number of variables and parameters, a model fitted to a single dataset will be prone to overfitting 85. In fact, even random data can generate a perfectly fitted model if the dataset is sufficiently large. There are two main ways to validate a model: (i) by bootstrapping using the same dataset and (ii) using an independent set of samples. In the first case, the dataset is partitioned into a training set and a test set. The model is generated using the training set, and its performance is validated against the test set. The number of misclassifications is then determined and provided that the response variable is binomial and corresponds to classes that could be labelled as ‘positive’ and ‘negative’, the errors can then be partitioned into false positives and false negatives. In the second case, the performance of the model is determined by validation with a separate dataset from another cohort, study or recruitment procedure.

Simply partitioning the dataset significantly decreases the size of the training set, resulting in a lower power of the approach. To this end, cross-validation procedures, typically a k-fold or leave-one-out (LOO) approach, are applied. In the case of LOO cross-validation, for each sample in the dataset, the remaining samples are used as a model training set. Any procedure such as specific filtering which uses the information of the response must only be applied to this training set and must not involve the given sample. The model is then applied to the sample, and the correctness of the prediction is recorded. Then, the next sample is considered, and the procedure is repeated until all samples have been assessed. In a k-fold cross-validation approach, the dataset is partitioned into k subsets, and for each subset, the same procedure as above is repeated: the subset is removed from the total dataset, and the remaining subsets are merged to form the training set 85. Although cross-validation maximizes the use of the information from the dataset, they do not result in a single model. Rather, a number of independent models are created for each replicate or fold, and the summary error rates are considered to be valid for the model constructed on the basis of the full dataset.

Despite the value of a cross-validation, the goodness of a model is directly related to its universal applicability. Even if a model performs extremely well for a dataset derived from a given experimental or clinical context, it may perform poorly in another patient cohort or for data collected by another technique. It is, therefore, imperative to test each model on independent data (external cross-validation). This procedure can only be realized correctly if the data used in cross-validation were not analyzed before creating the model; ideally, cross-validation should be carried out in a blinded manner. Even if the datasets for evaluation were not directly used for the model, mere knowledge of the results could bias the approach to model building. This phenomenon is known in statistics as ‘data snooping’ 86.

Nevertheless, some error rates (and counts of false positives and false negatives in the case of bimodal responses) are used to generate measures of the goodness of the model of which a number is available. In case of bimodal data, four distinct measures are of particular interest: sensitivity, specificity, positive predictive value (i.e. precision) and negative predictive value. The proportion of all correct classifications (100 minus the overall error rate) is known as the accuracy. In certain cases, further measures can reflect the true performance of the model much more reliably than the error rate alone. Accuracy or error rate is prone to the so-called accuracy paradox: if the categories are highly imbalanced (e.g. the prevalence of positive cases is very low), an algorithm uniformly assigning the ‘negative’ class in every prediction can have a lower overall error rate than a more meaningful model.

## Conclusions and outlook

Despite rapid progress over the last decade, vaccine development and biomarker research have not yet been linked in the area of TB research. Intensive development of HT biomarkers to provide surrogate end-points for vaccines in other therapeutic fields shows results that promise advancement also for TB vaccine design. In addition, biomarkers could help in stratification of study participants. Several vaccine candidates are considered to target adults with LTBI. Of these individuals, less than 10% will develop active TB. Selecting individuals with increased risk of active TB for a vaccine trial could reduce the number of study participants significantly and therefore also reduce the cost and duration of such vaccine trials. It is clear that further investigation combining experimental research, clinical studies and computational biology is required for the development of control measures against TB. It is hoped that the combined efforts of these different areas of expertise can accelerate the development of therapeutic approaches directed against this devastating disease.

## Conflict of interest statement

Stefan H.E. Kaufmann is coinventor of the TB vaccine VPM1002, scientific advisor of Aeras on TB vaccines and of Qiagen on TB biomarkers.
